# Inhibition of Vascular Inflammation by Apolipoprotein A-IV

**DOI:** 10.3389/fcvm.2022.901408

**Published:** 2022-06-30

**Authors:** Kate Shearston, Joanne T. M. Tan, Blake J. Cochran, Kerry-Anne Rye

**Affiliations:** ^1^Lipid Research Group, Faculty of Medicine, School of Medical Sciences, University of New South Wales, Sydney, NSW, Australia; ^2^Vascular Research Centre, Lifelong Health Theme, South Australian Health and Medical Research Institute, Adelaide, SA, Australia; ^3^Adelaide Medical School, The University of Adelaide, Adelaide, SA, Australia

**Keywords:** apolipoprotein A-IV, inflammation, high-density lipoproteins, endothelial cells, nuclear factor-kappaB, 3β-hydroxysteroid-Δ24 reductase

## Abstract

**Background:**

Apolipoprotein (apo) A-IV, the third most abundant apolipoprotein in human high density lipoproteins (HDLs), inhibits intestinal and systemic inflammation. This study asks if apoA-IV also inhibits acute vascular inflammation.

**Methods:**

Inflammation was induced in New Zealand White rabbits by placing a non-occlusive silastic collar around the common carotid artery. A single 1 mg/kg intravenous infusion of lipid-free apoA-IV or saline (control) was administered to the animals 24 h before collar insertion. The animals were euthanised 24 h post-collar insertion. Human coronary artery cells (HCAECs) were pre-incubated with reconstituted HDLs containing apoA-IV complexed with phosphatidylcholine, (A-IV)rHDLs, then activated by incubation with tumour necrosis factor (TNF)-α. Cell surface vascular cell adhesion molecule-1 (VCAM-1) and intercellular adhesion molecule-1 (ICAM-1) in the TNF-α-activated HCAECs was quantified by flow cytometry. VCAM-1, ICAM-1 and 3β-hydroxysteroid-Δ24 reductase (DHCR24) mRNA levels were quantified by real time PCR.

**Results:**

Apolipoprotein ApoA-IV treatment significantly decreased collar-induced endothelial expression of VCAM-1, ICAM-1 and neutrophil infiltration into the arterial intima by 67.6 ± 9.9% (*p* < 0.01), 75.4 ± 6.9% (*p* < 0.01) and 74.4 ± 8.5% (*p* < 0.05), respectively. It also increased endothelial expression of DHCR24 by 2.6-fold (*p* < 0.05). Pre-incubation of HCAECs with (A-IV)rHDLs prior to stimulation with TNF-α inhibited VCAM-1 and ICAM-1 protein levels by 62.2 ± 12.1% and 33.7 ± 5.7%, respectively. VCAM-1 and ICAM-1 mRNA levels were decreased by 55.8 ± 7.2% and 49.6 ± 7.9%, respectively, while DHCR24 mRNA expression increased by threefold. Transfection of HCAECs with DHCR24 siRNA attenuated the anti-inflammatory effects of (A-IV)rHDLs. Pre-incubation of TNF-α-activated HCAECs with (A-IV)rHDLs also inhibited nuclear translocation of the p65 subunit of nuclear factor-κB (NF-κB), and decreased IκBα phosphorylation.

**Conclusion:**

These results indicate that apoA-IV inhibits vascular inflammation *in vitro* and *in vivo* by inhibiting NF-κB activation in a DHCR24-dependent manner.

## Introduction

High-density lipoproteins (HDLs), and the main HDL apolipoprotein, apoA-I, have potent anti-inflammatory properties *in vitro* and *in vivo* (1–3). HDLs mediate these effects by inhibiting key steps in activation of the nuclear factor-κB (NF-κB) pathway, including nuclear translocation of the p65 subunit of NF-κB in multiple cell types (4–6). We have also reported that the anti-inflammatory properties of HDLs and apoA-I are related to their ability to increase expression of the anti-apoptotic and antioxidant enzyme, 3β-hydroxysteroid-Δ24 reductase (DHCR24) and induce the potentially cardioprotective enzyme, heme oxygenase-1 (5, 7).

Apolipoprotein A-IV, the third most abundant apolipoprotein in human HDLs, is a 46 kDa protein that is synthesised in the small intestine. It effluxes cholesterol from peripheral cells, including cholesterol-loaded macrophages, in the first step of the potentially anti-atherogenic reverse cholesterol transport pathway (8, 9), participates in the biogenesis of HDLs in an ATP binding cassette transporter A1- and lecithin:cholesterol acyltransferase-dependent manner (10) and has plaque stabilising properties (11).

ApoA-IV is a satiety factor and promotes thermogenesis in brown adipose tissue (12–14). It has anti-diabetic (15–17), antioxidant (18), anti-atherosclerotic (19, 20), anti-apoptotic (21) and anti-thrombotic properties (22) and inhibits intestinal inflammation and pro-inflammatory cytokine expression in animal models, as well as allergy-driven inflammation in humans and mice (20, 23, 24). The anti-inflammatory properties of apoA-IV are potentially clinically relevant and they have recently been identified as an independent risk marker of reduced inflammation in chronic kidney disease (25).

The present study asks if apoA-IV can inhibit acute vascular inflammation in New Zealand White (NZW) rabbits and, if so, whether it targets the same NF-κB- and DHCR24-related pathways as reported previously for HDLs and apoA-I. The results establish that extremely small amounts of apoA-IV markedly inhibit the vascular inflammation that is induced in these animals following insertion of a non-occlusive periarterial carotid collar. We also performed *in vitro* studies which established that the underlying mechanism of this anti-inflammatory effect is related, at least in part, to increased endothelial expression of DHCR24, and inhibition of NF-κB activation.

## Materials and Methods

### Animals

Male New Zealand White rabbits (approximately 2.5 kg) were obtained from the Institute of Medical and Veterinary Sciences (Gilles Plains, SA, Australia) and maintained on a normal chow diet. All procedures were approved by the Sydney South West Area Health Service Animal Welfare Committee (Project No. 2009/019A). The animals (*n* = 6/group) were randomised to receive a single intravenous (iv) infusion of either saline, lipid-free apoA-I [8 mg/kg (0.3 μmol/kg), positive control], or lipid-free apoA-IV [1 mg/kg, 0.02 μmol/kg)] into the left marginal ear vein 24 h before placing a non-occlusive, peri-arterial collar around the left common carotid artery (1). Prior to the procedure, the animals were sedated with subcutaneous acetylpromazine (0.5 mL/kg), then anaesthetised with inhaled isoflurane (4–5% for induction and 1.5–2% for maintenance). The animals were euthanised 24 h post-collar insertion and collared and non-collared carotid arteries were extracted for immunohistochemical analysis.

### Isolation of Apolipoprotein A-I and Rabbit High Density Lipoproteins

High density lipoproteins were isolated from pooled samples of autologously donated human plasma (Healthscope Pathology, Adelaide, SA, Australia) by sequential ultracentrifugation (1.063 < d < 1.21 g/mL) and delipidated using standard techniques (26). The resulting apoHDL was chromatographed on a Q-Sepharose Fast-Flow column and apoA-I was isolated as described (27, 28). The apoA-I preparations were judged to be >95% pure by electrophoresis on SDS-polyacrylamide PhastGels (GE Healthcare, Chicago, IL, United States) and Coomassie Blue staining.

For isolation of rabbit HDLs, plasma was adjusted to 1.25 g/mL with solid KBr and ultracentrifuged at 55,000 rpm for 16 h at 4°C, in a 70 Ti fixed angle rotor in an Optima LE-80K Ultracentrifuge (Beckman-Coulter Inc, Brea, CA, United States). The d < 1.25 g/mL fraction (600 μL) was injected onto two Superdex 200 columns connected in series to an AKTA FPLC system (GE Healthcare). The HDLs were eluted at a flow rate of 0.3 mL/min.

### Expression of Apolipoprotein A-IV

Apolipoprotein A-IV was cloned into the pET14b vector (with integral His-tag; Merck, Darmstadt, Germany), transformed into BL21 (pLysS) cells (Promega, Madison, WI, United States) and grown in Luria-Bertani (LB) culture media with ampicillin for selection of pET14b transformants. When the OD^600^ reached 0.6–0.8, protein expression was induced by incubation for 4 h with isopropyl β-D-1-thiogalactopyranoside (IPTG, 0.8 mM, Applichem, Darmstadt, Germany). The cells were pelleted, lysed with Bugbuster (Merck) and dialysed against 20 mM phosphate buffer containing 6 M urea, 0.3 M NaCl, and 20 mM imidazole (pH 8.0), then loaded onto 5 pre-equilibrated His-trap FF columns (5 mL, GE Healthcare) connected in series. ApoA-IV was eluted at 5 mL/min for 15 min with 20 mM phosphate buffer containing 6 M urea, 0.3 M NaCl and 500 mM imidazole (1–100% gradient) (pH 8.0). The apoA-IV was dialysed into TBS prior to removing the His-tag by incubation for 5 h at room temperature with thrombin (2 U/mg apoA-IV). The reaction was stopped by addition of Phosphatase Inhibitor Cocktail 2 (1 mL, Sigma-Aldrich, St Louis, MO, United States). The apoA-IV was dialysed into 20 mM Tris (pH 8.5), adjusted to 6 M with solid, deionised urea and loaded onto a pre-equilibrated Q-Sepharose Fast Flow column (GE Healthcare). ApoA-IV was eluted from the column at a flow rate of 3 mL/min with 20 mM Tris containing 6 M urea and a gradient of 0.5 M NaCl (pH 8.5): 0–40% for 5 min, 40–48% for 15 min, 48–70% for 15 min. The apoA-IV was dialysed against endotoxin-free PBS and added to 1% Triton X-114 (v/v) at 4°C for 30 min to remove residual endotoxin, then incubated at 37°C for 10 min and centrifuged (5,200 × *g*) for 1 h. The aqueous layer (containing apoA-IV) was removed, and the process repeated three times. Residual Triton X-114 was removed from the apoA-IV by incubation for 2 h at 4°C with Bio beads (Bio-Rad, Hercules, CA, United States). The apoA-IV was concentrated (Amicon Ultra -15 centrifugal filter, Billerica, MA, United States) and filtered (0.22 μM). Endotoxin levels were tested using an EndoSafe PTS-Reader and 0.1–10 EU/mL PTS cartridges (Charles River, Wilmington, MA, United States) and found to be <0.5 EU/mL.

### Preparation of Discoidal Reconstituted HDLs

Discoidal rHDLs containing palmitoyl-2-linoleoyl-*sn*-glycero-3-phosphatidylcholine (PLPC, Avanti Polar Lipids, Alabaster, AL, United States) and apoA-I [initial PLPC/apoA-I molar ratio 100/1, (A-Ir)HDLs] or apoA-IV [initial PLPC/apoA-IV molar ratio 150/1, (A-IV)rHDLs] were prepared by the cholate dialysis method (29). The rHDLs were dialysed extensively against endotoxin-free PBS before use. Particle sizes were determined by non-denaturing gradient gel electrophoresis (30). Cross-linking with the homo-bifunctional cross-linking agent bis (sulfosuccinimidylsuccinate) (BS) was performed as described (31, 32).

### Cell Culture

Human coronary artery endothelial cells (HCAECs, passages 2 to 6) were maintained in MesoEndo media (Cell Applications, San Diego, CA, United States). The cells, in either 12-well (1 × 10^5^ cells/well for mRNA quantification) or 6-well plates (2 × 10^5^ cells/well for protein extraction) were pre-incubated for 16 h with discoidal (A-I)rHDLs, discoidal (A-IV)rHDLs, isolated rabbit HDLs or PBS. After removal of the rHDLs or HDLs, the cells were incubated for 5 h at 37°C with TNF-α (final concentration 0.2 ng/mL), then subjected to flow cytometry or total RNA isolation. For measurement of the p65 subunit of NF-κB and IκBα, the cells were incubated with TNF-α (final concentration 0.2 ng/mL) for 20 min.

### Knockdown of 3β-Hydroxysteroid-Δ24 Reductase

Human coronary artery endothelial cells (0.5 × 10^5^ cells/well) were plated into 24-well plates and transfected at 37°C for 7 h with either DHCR24 siRNA (Santa Cruz, Dallas, TX, United States; sc-60531) or scrambled control siRNA (Santa Cruz; sc-37007) using 15 pmol of siRNA.

### Flow Cytometry

Human coronary artery endothelial cells were plated into 24-well (0.5 × 10^5^ cells/well) and incubated at 4°C for 30 min with an ICAM-1 antibody (1:5; CD54 FITC, Beckman-Coulter), a VCAM-1 antibody (1:500; CD106 PeCy5 510C9, BD Bioscience, Franklin Lakes NJ, United States) or PBS containing 10% (v/v) heat-inactivated fetal bovine serum (FBS). An isotype control (PECy5 mouse IgG1, BD Bioscience and IgG1 FITC, Beckman-Coulter) was used to confirm the specificity of the fluorescent labelling. Cells incubated in the absence of TNF-α were used to ascertain background expression of ICAM-1 and VCAM-1.

### Western Blotting

Human coronary artery endothelial cells were rinsed with PBS and nuclear and cytoplasmic extracts were prepared (NE-PER, Pierce, Rockford, Ill). The extracted proteins were quantified (BCA assay), loaded onto 3–14% Bis-Tris gels (Life Technologies, Carlsbad, CA, United States), electrophoresed for 45 min, transferred to nitrocellulose membranes and blocked with 10% (w/v) skim milk. Phosphorylated and total IκBα, the p65 subunit of NF-κB and β-actin were detected with mouse anti-human IκBα (1:1000, Cell Signalling), rabbit anti-human phospho-IκBα (1:1000, Cell Signalling Technology, Danvers, MA, United States), rabbit anti-human p65 (1:500, Santa Cruz Biotechnology, Dallas, TX, United States) and mouse anti-human β-actin (1:3000) monoclonal antibodies (Abcam, Cambridge, United Kingdom). Goat anti-rabbit or goat anti-mouse IgG-HRP (Santa-Cruz) were used as secondary antibodies. The membranes were developed using ECL Plus (GE Healthcare) and imaged with a Chemidoc imager (Bio-Rad).

### Plasma Lipid and Lipoprotein Analysis

All analyses were carried out in triplicate on a Hitachi 902 autoanalyser (Roche Diagnostics, Mannheim, Germany). Protein concentrations were measured using the bicinchoninic acid assay (33). Phospholipid and unesterified cholesterol concentrations were measured enzymatically (34, 35). Total cholesterol concentrations were measured using commercial kits (Roche Diagnostics). ApoA-I was measured immunoturbidometrically using a goat anti-human apoA-I antibody (Calbiochem, San Diego, CA, United States) (36). ApoA-IV was quantified using a commercially available ELISA kit (Millipore, Burlington, MA, United States).

### Immunohistochemistry

Carotid artery sections were dehydrated and embedded in paraffin, sectioned (5 μm), and mounted on glass slides. The sections were then incubated overnight at 37°C prior to antigen retrieval (Target Retrieval Buffer (pH 9.0), Dako, Glostrup, Denmark). After blocking endogenous peroxidase activity with Peroxidase block (Envision Mouse Kit, Dako), the sections were incubated with 10% (v/v) rabbit serum to block non-specific binding and stained with mouse anti-rabbit CD18 (1:200; AbD Serotec, Raleigh, NC, United States), mouse anti-rabbit VCAM-1 (1:200), mouse anti-rabbit ICAM-1 (1:200) (provided Dr M. Cybulsky, University of Toronto) monoclonal antibodies or a goat anti-human polyclonal antibody against DHCR24 (1:200) (Santa-Cruz). The sections were rinsed, incubated with appropriate HRP-conjugated secondary antibodies (Envision Mouse Kit, Dako), then incubated with 3,3-diaminobenzidine solution (Envision Mouse Kit, Dako) and counter-stained with Haematoxylin and Eosin. Images were obtained using a Zeiss Imager M1 microscope with an attached AxioCam MRc5 at 40 × magnification. Staining was quantified using ImageJ.^1^ The threshold for positive staining was determined by a blinded observer and applied to all sections. To account for variations in carotid artery size, the number of pixels staining positive for endothelial VCAM-1 and ICAM-1 was divided by the endothelial circumference and the results were expressed as image units. CD18-positive and DHCR24-positive staining was expressed as % total intima/media cross-sectional area.

### Real Time PCR

Total RNA was isolated with Trizol and reverse transcribed using an iScript cDNA synthesis kit (Bio-Rad) and a Mastercycler thermocycler (Eppendorf, Hamburg, Germany). Real time PCR was performed using iQ SYBR Green Supermix (Bio-Rad) in an iCycler iQ Real-Time thermocycler. Quantitation of gene expression was assessed by the ΔΔCt method with β-2 microglobulin (B2 M), hypoxanthine phosphoribosyltransferase 1 (HPRT-1), and β-actin as housekeeping genes. Primers are shown in Supplementary Table 1.

### Statistical Analysis

All statistical analyses were performed using GraphPad Prism Version 5.0 d for Mac (GraphPad Software, San Diego, CA United States).^2^ Significant differences between data sets were determined using a two-tailed, unpaired Student’s *t*-test or one-way ANOVA with Dunnett’s post-test, with *p* < 0.05 considered significant. The normality of all data was determined with the Kolmogorov-Smirnov test and α = 0.05 considered to be normally distributed. All results are presented as mean ± SEM unless otherwise specified.

## Results

### A Single Infusion of Lipid-Free Apolipoprotein A-IV Inhibits Peri-Arterial Collar-Induced Vascular Inflammation in New Zealand White Rabbits

**FIGURE 1:**
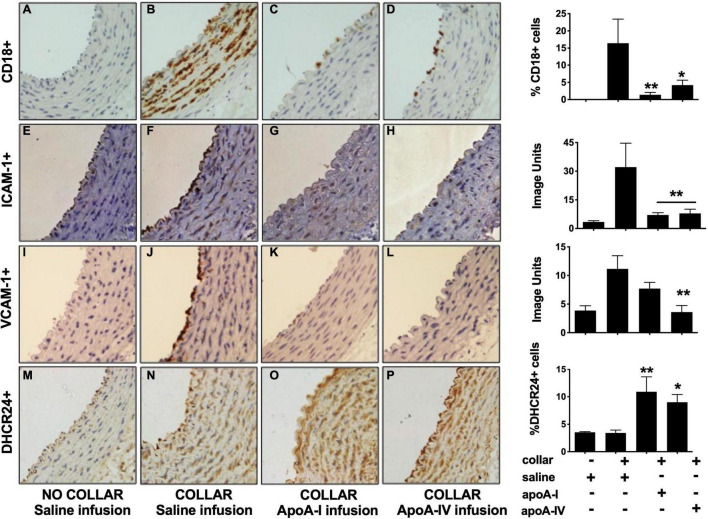
Apolipoprotein A-IV inhibits acute vascular inflammation in NZW rabbit carotid arteries. NZW rabbits (*n* = 6/group) were infused iv with saline [Panels **(A,B,E,F,I,J,M,N)**], apoA-I [Panels **(C,G,K,O)**; 8 mg/kg iv] or apoA-IV [Panels **(D,H,L,P)**; 1 mg/kg iv] 24 h prior to inserting a non-occlusive peri-arterial collar around the left common carotid artery. The animals were euthanised 24 h post-collar insertion. Sections from the non-collared [Panels **(A,E,I,M)**] and the collared carotid arteries were stained for neutrophils [CD18, Panels **(B–D)**], ICAM-1 [Panels **(F–H)**], VCAM-1 [Panels **(J–L)**] and DHCR24 [Panels **(N–P)**]. Representative immunostained images as shown. *p < 0.05, **p < 0.01.

A non-occlusive collar was placed around the common carotid artery of NZW rabbits (*n* = 6/group) 24 h after administration of a single infusion of apoA-IV (1 mg/kg iv), saline (negative control) or apoA-I (8 mg/kg, positive control). As reported previously, neutrophils (CD18^+^ cells) were not detected in the non-collared arteries in the saline-infused animals (Figure 1A) (1). The arteries also had low levels of constitutive ICAM-1 (Figure 1E) and VCAM-1 (Figure 1I) expression.

The collar induced an acute inflammatory response that was accompanied by neutrophil infiltration into the intima-media (Figure 1B) and increased endothelial expression of ICAM-1 (Figure 1F) and VCAM-1 (Figure 1J). A single 8 mg/kg iv infusion of lipid-free apoA-I 24 h prior to collar insertion reduced collar-mediated CD18^+^ cell infiltration from 16.4 ± 6.7% (Figure 1B) to 1.4 ± 0.7% of the total intima-media area (Figure 1C, *p* < 0.01). A single 1 mg/kg iv infusion of apoA-IV similarly decreased the area staining positive for CD18^+^ cells from 16.4 ± 6.7% (Figure 1B) to 4.2 ± 1.4%, (Figure 1D, *p* < 0.05).

The collar-mediated increase in endothelial ICAM-1 expression was reduced from 32.1 ± 12.5 image units (Figure 1F) to 7.1 ± 1.2 image units (Figure 1G) in the animals that were infused with apoA-I, and to 7.9 ± 2.2 image units (Figure 1H) in the animals that were infused with apoA-IV (*p* < 0.01 for both). The collar-induced increase in endothelial VCAM-1 expression was attenuated from 11.1 ± 2.3 image units (Figure 1J) in the saline-infused animals to 7.7 ± 1.1 image units (Figure 1K) in the animals that were infused with lipid-free apoA-I, and to 3.6 ± 1.1 image units (Figure 1L) in the apoA-IV-infused animals, (*p* < 0.01 for both).

Insertion of a carotid collar did not alter DHCR24 protein expression in the intima-media of the saline-infused animals relative to what was observed for the non-collared arteries (Figures 1M,N). This is consistent with what has been reported previously (37). Expression of DHCR24 in the intima-media, by contrast, increased from 3.4 ± 0.5% (Figure 1M) in the collared, saline-infused animals to 10.9 ± 2.7% of the total area in the animals that were infused with apoA-I (Figure 1O, *p* < 0.01), and to 9.0 ± 1.4% in the animals that were infused with apoA-IV (Figure 1P, *p* < 0.05).

### A Single Apolipoprotein A-IV Infusion Does Not Affect Plasma Lipids or High Density Lipoprotein Size and Composition

Plasma apoA-I, apoA-IV, phospholipid, unesterified cholesterol, total cholesterol and phospholipid levels did not differ between the three groups of rabbits at baseline (Supplementary Table 2). At 24 h post-infusion (i.e., at the time of collar insertion) plasma lipid, apoA-I and apoA-IV levels in the rabbits that received saline, apoA-IV and apoA-I were comparable. There was also no difference in plasma lipid, apoA-I and apoA-IV levels at 48 h after the infusion of apoA-IV, apoA-I or saline.

HDLs were isolated from plasma as described in section “Materials and Methods” and, given that rabbits are deficient in apoA-II, the composition of the preparations was determined based on the assumption that apoA-I is the predominant apolipoprotein (38). HDL composition did not differ significantly in the animals that were infused with saline, apoA-IV or apoA-I (Supplementary Table 3). This is consistent with what has been reported previously for HDLs isolated from saline- and apoA-I-infused rabbits (39). The apoA-I and apoA-IV infusions also had no effect on HDL particle size (Supplementary Table 3).

### A Single Apolipoprotein A-IV Infusion Does Not Improve the Anti-inflammatory Function of High Density Lipoproteins in New Zealand White Rabbits

**FIGURE 2:**
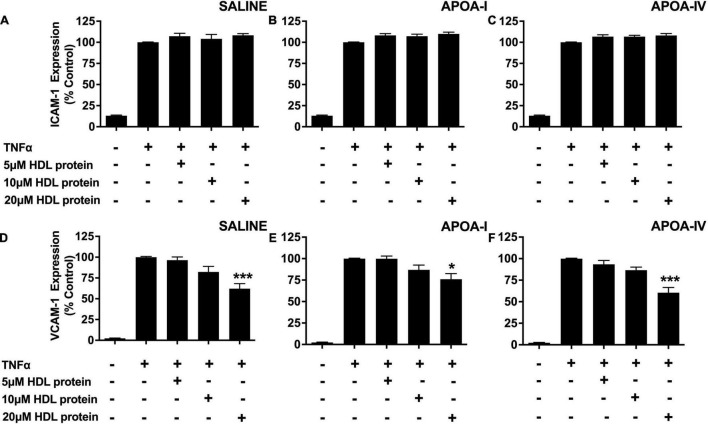
A single apoA-IV infusion does not alter the anti-inflammatory properties of rabbit HDLs. HDLs were ultracentrifugally isolated from NZW rabbits that had been treated with saline [Panels **(A,D)**], lipid-free apoA-I [Panels **(B,E)**] or lipid-free apoA-IV [Panels **(C,F)**] as described in the legend to Figure 1. The isolated HDLs (final apoA-I concentration: 5, 10, or 20 μM) were incubated at 37°C for 16 h with HCAECs prior to stimulation with TNF-α (0.2 ng) for 5 h at 37°C. ICAM-1 and VCAM-1 protein expression was quantified by flow cytometry. Data points represent mean ± SEM of three independent experiments with three replicates/experiment. *p < 0.05, ***p < 0.001.

The anti-inflammatory properties of the HDLs that were isolated from the saline-, apoA-I- and apoA-IV-infused rabbits were also evaluated in TNF-α-activated HCAECs as described in section “Materials and Methods.” The isolated HDLs did not inhibit ICAM-1 expression in TNF-α-activated HCAECs, irrespective of whether the animals were infused with saline, lipid-free apoA-I, or lipid-free apoA-IV (Figures 2A–C).

The isolated HDLs from the saline-, apoA- I-, and apoA-IV -infused rabbits, by contrast, inhibited VCAM-1 expression in a concentration dependent manner (Figures 2D–F). At an HDL protein concentration of 20 μM, the HDLs from the saline-infused rabbits decreased VCAM-1 protein levels by 42.9 ± 7.3% (Figure 2D, *p* < 0.001), compared to 24 ± 6.5% for the rabbits that were infused with apoA-I (Figure 2E, *p* < 0.05) and by 39.5 ± 6.0% for the rabbits that were infused with apoA-IV (Figure 2F, *p* < 0.001).

As the reduction in VCAM-1 expression was similar irrespective of whether the rabbits were infused with saline, apoA-I or apoA-IV, it was concluded that treatment with apoA-IV does not improve the anti-inflammatory function of HDLs.

### (A-IV)rHDLs Inhibit Intercellular Adhesion Molecule 1 and Vascular Cell Adhesion Molecule 1 Expression in Human Coronary Artery Endothelial Cells More Effectively Than (A-I)rHDLs

Although apoA-IV inhibits intestinal inflammation and decreases atherosclerosis in mice (20, 23), it has a low affinity for lipid, with up to 98% of the total apoA-IV in fasted human plasma being present in the lipid-free form after ultracentrifugation (40). This indicates that most of the apoA-IV may have dissociated from the rabbit HDLs during the isolation procedure, thus explaining why the HDL preparations from the apoA-IV-treated rabbits inhibited VCAM-1 expression in HCAECs to the same extent as the isolated HDLs from the apoA-I-treated rabbits (Figure 2).

**FIGURE 3:**
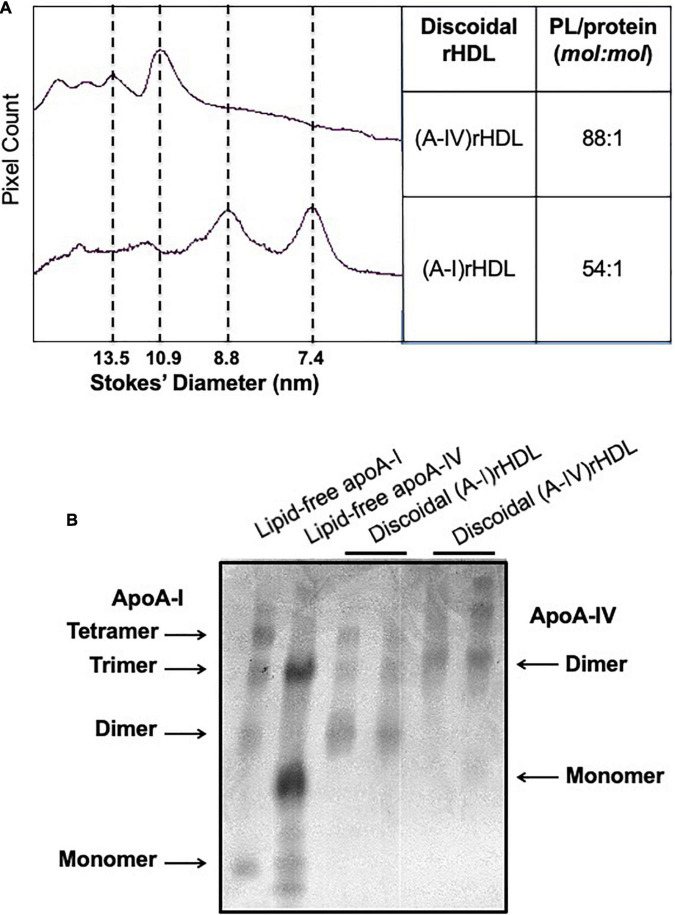
Size and composition of discoidal (A-IV)rHDLs and discoidal (A-I)rHDLs. Discoidal (A-IV)rHDLs and (A-I)rHDLs were prepared by the cholate dialysis method and analysed in terms of size and composition [Panel **(A)**]. The discoidal rHDLs were then cross-linked with BS and their migration was compared with that of cross-linked lipid-free apoA-I and apoA-IV [Panel **(B)**].

To ascertain whether apoA-IV is able to inhibit ICAM-1 and VCAM-1 in endothelial cells *in vitro*, discoidal (A-IV)rHDLs were prepared by complexing lipid-free apoA-IV with PLPC. The discoidal (A-IV)rHDLs consisted of a major population of particles 10.9 nm in diameter and three larger, minor populations of particles (Figure 3A). Discoidal (A-I)rHDLs were used as a positive control and consisted predominantly of particles 8.8 and 7.4 nm in diameter (Figure 3A). The PLPC/apolipoprotein molar ratio of the (A-IV)rHDLs and (A-I)rHDLs were 88/1 and 54/1, respectively (Figure 3A). As judged by covalent cross-linking, the (A-I)rHDL and (A-IV)rHDL preparations consisted of populations of particles with two, three and four apolipoprotein molecules/particle (Figure 3B).

**FIGURE 4:**
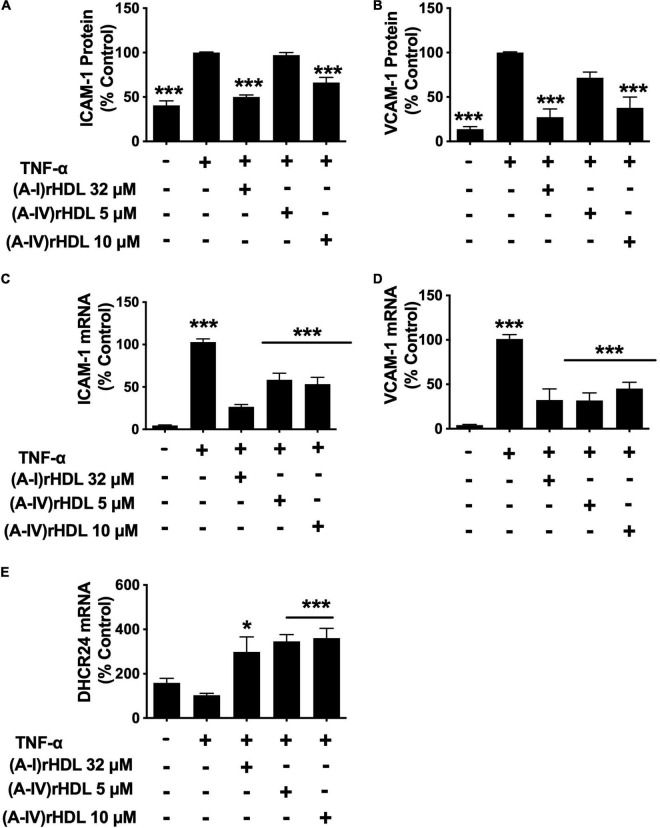
(A-IV)rHDLs inhibit ICAM-1, VCAM-1 expression and increase DHCR24 expression in TNF-α-stimulated HCAECs. HCAECs were incubated for 16 h with (A-I)rHDLs (final apoA-I concentration 32 μM) and (A-IV)rHDLs (final apoA-IV concentrations 5 and 10 μM). The rHDLs were then removed and the cells were incubated for a further 5 h with TNF-α (final concentration 0.2 ng/mL). ICAM-1 [Panel **(A)**] and VCAM-1 [Panel **(B)**] protein expression was quantified by flow cytometry. ICAM-1 [Panel **(C)**], VCAM-1 [Panel **(D)**], and DHCR24 [Panel **(E)**] mRNA levels were quantified by qPCR. The mean ± SEM of three independent experiments, each performed in triplicate are shown. *p < 0.05, ***p < 0.005.

Pre-incubation of HCAECs with the discoidal (A-I)rHDLs at a final apoA-I concentration of 32 μM prior to stimulation with TNF-α, inhibited cell surface ICAM-1 levels by 50 ± 2% (Figure 4A) and VCAM-1 levels by 73 ± 9% (Figure 4B) (*p* < 0.001 for both compared to TNF-α-stimulated HCAECs). Pre-incubation of the HCAECs with discoidal (A-IV)rHDLs at a final apoA-IV concentration of 5 μM did not inhibit ICAM-1 or VCAM-1 expression significantly. Pre-incubation with discoidal (A-IV)rHDLs at a final apoA-IV concentration of 10 μM, by contrast, inhibited ICAM-1 and VCAM-1 levels by 34 ± 3% (Figure 4A) and 62 ± 12% respectively (Figure 4B) (*p* < 0.001 for both compared to TNF-α-stimulated HCAECs).

The reduction in ICAM-1 and VCAM-1 protein levels was accompanied by a decrease in their respective mRNA levels. Pre-incubation of HCAECs with discoidal (A-I)rHDLs at a final apoA-I concentration of 32 μM inhibited ICAM-1 (Figure 4C) and VCAM-1 mRNA levels (Figure 4D) by 76 ± 3% and 69 ± 12% respectively (*p* < 0.001 for both compared with TNF-α-stimulated HCAECs). Pre-incubation of HCAECs with discoidal (A-IV)rHDLs at a final apoA-IV concentration of 5 or 10 μM decreased ICAM-1 mRNA levels by 44 ± 8% and 50 ± 8%, respectively (Figure 4C, *p* < 0.001 for both compared with TNF-α-activated HCAECs), while VCAM-1 mRNA levels were decreased by 68 ± 9% and 55 ± 7%, respectively (Figure 4C) (*p* < 0.001 for both). These results are consistent with rHDLs that contain apoA-IV being more effective on a per particle basis than (A-I)rHDLs at inhibiting ICAM-1 and VCAM-1 mRNA levels and transcription in TNF-α-activated HCAECs.

Pre-incubation of HCAECs with discoidal (A-I)rHDLs (final apoA-I concentration 32 μM) prior to stimulation with TNF-α also increased DHCR24 mRNA levels by 196 ± 68% (Figure 4E, *p* < 0.05). This is consistent with what has been reported previously (5). Similarly, pre-incubation of TNF-α-stimulated HCAECs with discoidal (A-IV)rHDLs at final apoA-IV concentrations of 5 or 10 μM increased DHCR24 mRNA levels by 244 ± 30% and 257 ± 44%, respectively (Figure 4E, *p* < 0.001 for both).

### (A-IV)rHDLs Inhibit Nuclear Factor-kappaB Activation in Human Coronary Artery Endothelial Cells

**FIGURE 5:**
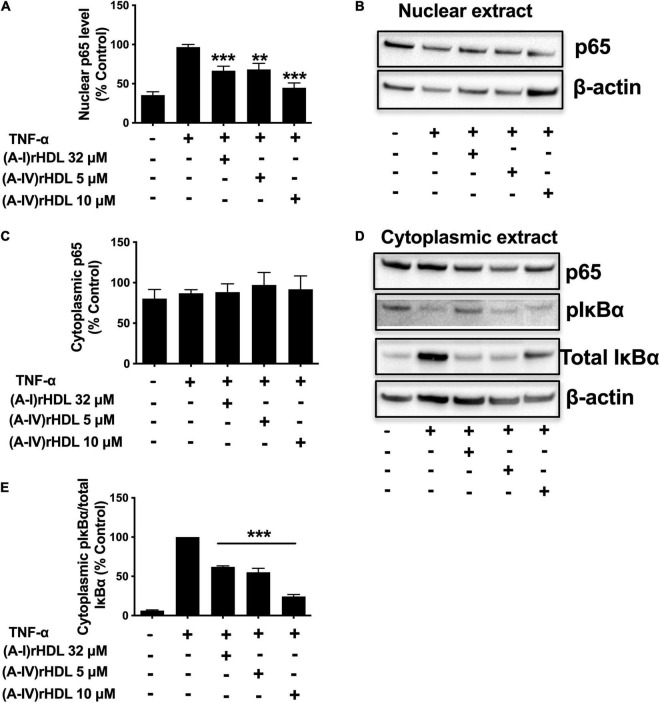
(A-IV)rHDL inhibits NF-κB activation in TNF-α-stimulated HCAECs. HCAECs were incubated for 16 h at 37°C with (A-I)rHDLs (final apoA-I concentration 32 μM) or (A-IV)rHDLs (final A-IV concentration 5 and 10 μM). The rHDLs were removed and the cells were incubated for 20 min at 37°C with TNF-α (0.2 ng/mL). Nuclear extracts were isolated and subjected to immunoblotting using β-actin as a loading control. Nuclear p65 protein levels **(A,B)** and cytoplasmic levels of p65 and phosphorylated IκBα (pIκBα), **(C,D)** are shown. **(E)** Shows the ratio of cytoplasmic pIκBα/IκBα. Results represent mean ± SEM of three independent experiments, each performed in triplicate. **p < 0.01, ***p < 0.001.

To ascertain if (A-IV)rHDLs inhibit ICAM-1 and VCAM-1 expression in HCAECs *via* the canonical NF-κB pathway, nuclear p65, as well as cytoplasmic levels of total and phosphorylated IκBα were quantified in TNF-α-activated HCAECs. Incubation with TNF-α increased HCAEC nuclear p65 levels (Figures 5A,B). Pre-incubation of the cells with discoidal (A-I)rHDLs (final apoA-I concentration 32 μM) prior to stimulation with TNF-α, reduced nuclear p65 protein levels by 34 ± 6% compared to cells incubated with TNF-α alone (Figures 5A,B), (*p* < 0.001). Pre-incubation of TNF-α-stimulated HCAECs with discoidal (A-IV)rHDLs at final apoA-IV concentrations of 5 or 10 μM inhibited nuclear p65 levels by 33 ± 8% (*p* < 0.01) and 56 ± 6% (*p* < 0.001), respectively, compared to cells incubated with TNF-α alone) (Figures 5A,B). Cytoplasmic p65 levels were not affected by pre-incubation with discoidal (A-I)rHDLs or discoidal (A-IV)rHDLs, or by incubation with TNF-α (Figures 5C,D).

Incubation with TNF-α markedly increased the phosphorylated IκBα/total IκBα ratio in HCAECs (Figures 5D,E). Pre-incubation of HCAECs with (A-I)rHDLs prior to TNF-α-stimulation reduced the phosphorylated IκBα/total IκBα ratio by 38.1 ± 1.3% (Figures 5D,E, *p* < 0.001). This is consistent with what has been reported previously (4). Pre-incubation of TNF-α-stimulated HCAECs with discoidal (A-IV)rHDLs at final concentrations of 5 or 10 μM reduced the phosphorylated IκBα/total IκBα ratio by 45.0 ± 5.0% and 75.7 ± 2.4%, respectively (Figures 5D,E) (*p* < 0.001 for both). Taken together, these results indicate that discoidal (A-IV)rHDLs decrease cytokine-induced inflammation in HCAECs by inhibiting NF-κB activation.

### (A-IV)rHDLs Inhibit Intercellular Adhesion Molecule 1 and Vascular Cell Adhesion Molecule 1 Expression in Tumour Necrosis Factor Alpha Stimulated Human Coronary Artery Endothelial Cells in a 3β-Hydroxysteroid-Δ24 Reductase -Dependent Manner

**FIGURE 6:**
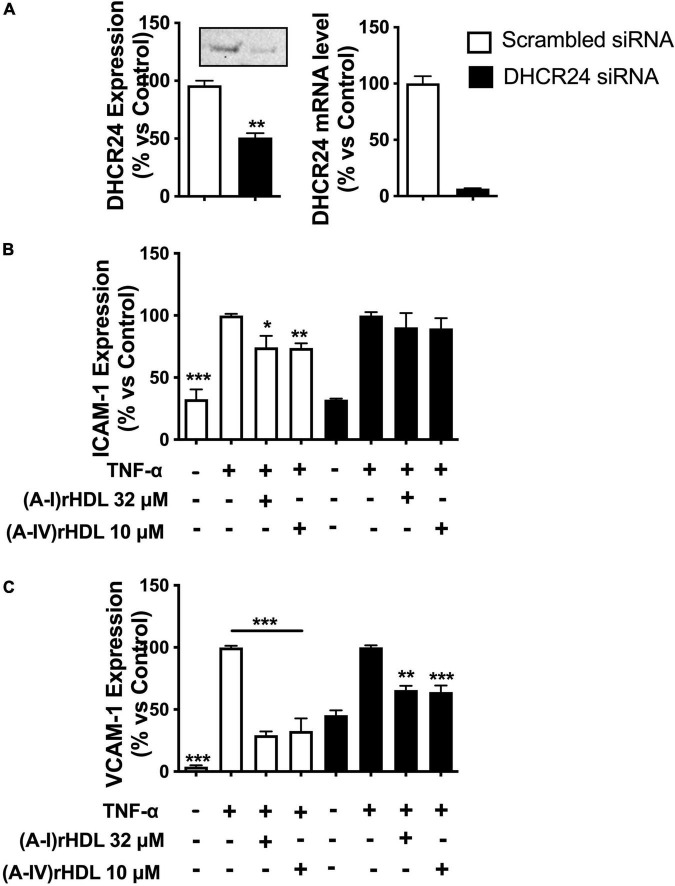
(A-IV)rHDLs inhibit TNF-α-induced ICAM-1 and VCAM-1 expression in HCAECs in a DHCR24-dependent manner. HCAECs were transfected with DHCR24 or scrambled siRNA then pre-incubated at 37°C for 16 h with (A-I)rHDLs (final apoA-I concentration 32 μM) or (A-IV)rHDLs (final apoA-IV concentration 10 μM). The rHDLs were removed and the cells were stimulated for 5 h with TNF-α (final concentration 0.2 ng/mL). The reduction in DHCR24 protein and mRNA levels in the transfected HCAECs was quantified by Western blotting and qPCR [Panel **(A)**]. ICAM-1 [Panel **(B)**] and VCAM-1 [Panel **(C)**] protein levels were quantified in the TNF-α-activated HCAECs by flow cytometry. Results represent the mean ± SEM of three independent experiments, each performed in triplicate. *p < 0.05, **p < 0.01, ***p < 0.001 vs. TNF-α-stimulated cells.

Transfection of HCAECs with DHCR24 siRNA reduced DHCR24 protein and mRNA levels by 45 ± 4 (*p* < 0.01) and 93 ± 6%, respectively, relative to HCAECs transfected with scrambled siRNA (Figure 6A).

Incubation of the scrambled, siRNA-transfected HCAECs with TNF-α significantly increased ICAM-1 protein expression (Figure 6B, open bars) (*p* < 0.001). Pre-incubation of the scrambled, siRNA-transfected HCAECs with discoidal (A-I)rHDLs (final apoA-I concentration 32 μM) or discoidal (A-IV)rHDLs (final apoA-IV concentration 10 μM), prior to stimulation with TNF-α, inhibited the cytokine-induced increase in ICAM-1 expression by 26 ± 9% (*p* < 0.05) and 26 ± 4% (*p* < 0.01), respectively (Figure 6B, open bars). ICAM-1 expression was not inhibited in HCAECs that were transfected with DHCR24 siRNA and pre-incubated with discoidal (A-IV)rHDLs or discoidal (A-I)rHDLs prior to stimulation with TNF-α (Figure 6B, closed bars).

Pre-incubation of scrambled siRNA-transfected-HCAECs with discoidal (A-I)rHDLs and discoidal (A-IV)rHDLs prior to stimulation with TNF-α inhibited the cytokine-induced increase in VCAM-1 expression by 71 ± 3% (Figure 6C, open bars) and 67 ± 10% (Figure 6C, open bars), respectively (*p* < 0.001 for both compared with TNF-α-stimulated cells). For HCAECs that were transfected with DHCR24 siRNA, and pre-incubated with (A-I)rHDLs prior to activation with TNF-α, the cytokine-induced increase in VCAM-1 expression was inhibited by 34 ± 3% (Figure 6C, *p* < 0.01), and by 36 ± 5% for the cells that were pre-incubated with (A-IV)rHDLs (Figure 6C, *p* < 0.001).

## Discussion

**FIGURE 7:**
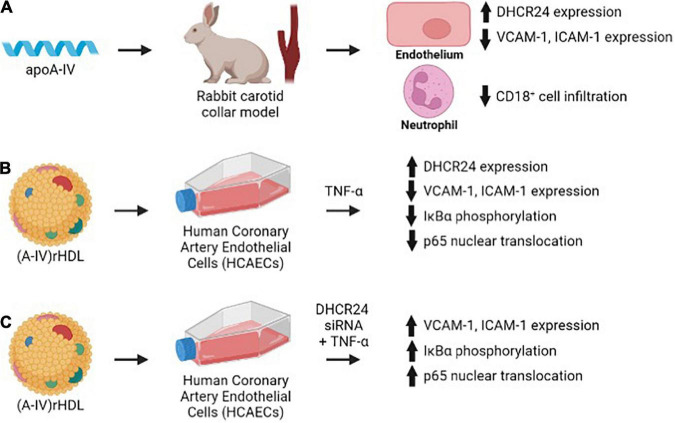
Lipid-free and lipid-associated apoA-IV reduces acute vascular inflammation by inhibiting endothelial ICAM-1 and VCAM-1 expression **(A,B)** and NF-κB activation in a DHCR24-dependent manner **(C)**.

This study shows for the first time that apoA-IV is a more effective inhibitor of acute vascular inflammation in NZW rabbits than apoA-I. This is demonstrated by the ability of a single 1 mg/kg (2 × 10^–8^ mol/kg) iv injection of apoA-IV to reduce collar-induced neutrophil infiltration into the carotid artery, inhibit endothelial expression of ICAM-1 and VCAM-1 and increase expression of the anti-apoptotic and antioxidant enzyme, DHCR24, in NZW rabbits to a comparable extent as a single 8 mg/kg (28 × 10^–8^ mol/kg) iv injection of apoA-I (Figure 7A) (2). The results also establish that discoidal (A-IV)rHDLs reduce ICAM-1 and VCAM-1 expression in TNF-α-activated HCAECs by inhibiting NF-κB activation in a DHCR24-dependent manner (Figures 7B,C).

These outcomes extend previous studies in which lipid-free apoA-IV reduced inflammation in a mouse model of colitis, inhibited histamine release from basophils in patients with allergic rhinitis and reduced TNF-α secretion from human monocytes following stimulation with lipopolysaccharide (20, 23, 41). Importantly, apoA-I was unable to recapitulate the anti-inflammatory properties of apoA-IV in any of these studies (20, 23, 41), thus identifying apoA-IV as a unique, anti-inflammatory agent with therapeutic potential.

As the HDLs that were isolated from apoA-IV-treated NZW rabbits did not inhibit cell surface ICAM-1 or VCAM-1 expression in TNF-α-activated HCAECs to a greater extent than what was observed for saline-infused NZW rabbits, the anti-inflammatory properties of apoA-IV cannot be explained in terms of improved HDL function. However, it is possible that the anti-inflammatory properties of the endogenous HDLs were improved immediately post-infusion, but that this was no longer evident at 48 h post-infusion, when the animals were euthanised prior to isolation of HDLs. This is consistent with what we have reported previously, where there was a transient improvement in the anti-inflammatory properties of HDLs isolated from rabbits at 5 min, but not at 6 h, after a single infusion of apoA-I (39). It is also possible that the association of apoA-IV with endogenous rabbit HDLs was disrupted during the ultracentrifugal isolation process, such that the isolated HDLs from the apoA-IV-treated rabbits were not selectively enriched with this apolipoprotein.

The lack of a sustained effect of apoA-IV on HDL function is further supported by the fact that turnover of this apolipoprotein in human plasma is 8.7 mg/kg/day (42), and is likely to be considerably faster than that in NZW rabbits. Given that the animals weighed 2–3 kg and apoA-IV was administered at a dose of 1 mg/kg, it follows that the amount of human apoA-IV remaining in the plasma compartment of these animals after 48 h was most likely minimal.

Structural differences between apoA-IV and apoA-I may have contributed to the superior *in vivo* anti-inflammatory properties of apoA-IV. Both apoA-IV and apoA-I contain 22 amino acid amphipathic α-helical repeats that are disrupted by conserved proline residues (43). Both apolipoproteins also contain Class Y and atypical Class A α-helices (43). However, the class Y α-helices that predominate in apoA-IV, and may contribute to its low affinity for lipid relative to apoA-I, favour partitioning into cell membranes at the level of the phospholipid head groups, rather than the phospholipid acyl chains, which is where apoA-I is located (43, 44). As studies of apoA-I mimetic peptides have indicated that their anti-inflammatory properties are enhanced by association with cell membrane phospholipid headgroups rather than acyl chains, it follows that differential partitioning may have contributed to the enhanced anti-inflammatory properties of apoA-IV relative to those of apoA-I (45–47).

One of the main aims of this study was to obtain an insight into the mechanism by which apoA-IV inhibits vascular inflammation. As lipid-free apoA-I and (A-I)rHDLs decrease expression of ICAM-1 and VCAM-1 by inhibiting NF-κB activation in a DHCR24-dependent manner (5, 39), this pathway was investigated in the current study. This was achieved be evaluating the ability of (A-IV)rHDLs to reduce the activity of IκB kinase, reduce the ratio of phosphorylated IκBα to total IκBα, and reduce nuclear localisation of the p65 subunit of NF-κB (4, 5, 48, 49).

The results of these *in vitro* studies confirmed that (A-IV)rHDLs inhibit all of these steps in the NF-κB pathway, and additionally increase expression of the anti-oxidant and anti-apoptotic enzyme DHCR24. Interestingly apoA-IV, both in a lipid-free form *in vivo* and as a constituent of discoidal (A-IV)rHDLs *in vitro*, increased DHCR24 protein and mRNA levels at much lower concentrations than apoA-I. However, the ability of apoA-IV to inhibit VCAM-1, but not ICAM-1, *in vitro* was only partly dependent on DHCR24. This suggests that pathways other than inhibition of NF-κB must also contribute to the anti-inflammatory properties of discoidal (A-IV)rHDLs.

An alternate pathway by which discoidal (A-IV)rHDLs may inhibit inflammation in endothelial cells is *via* interaction with scavenger receptor class B type 1 (SR-B1). Discoidal (A-I)rHDLs have been reported to interact with SR-B1 and the SR-B1 adaptor protein, PDZK1, to increase DHCR24 expression and induce the cytoprotective protein, heme-oxygenase 1 (37). As lipid-free apoA-IV and discoidal (A-IV)rHDLs both interact with SR-B1 (10), it is possible that they may inhibit inflammation in endothelial cells *via* this pathway.

Another explanation for the anti-inflammatory properties of (A-IV)HDLs may be related to their ability to increase bioavailability of the vasodilator nitric oxide (NO) in endothelial cells. This possibility is related to the observation that inhibition of NO production is associated with upregulation of endothelial monocyte chemoattractant protein-1 (MCP-1) and VCAM-1 expression *via* the NF-κB pathway (50, 51). ApoA-IV-containing HDLs may therefore protect against these adverse effects by inhibiting MCP-1 and VCAM-1 expression as well as activation of NF-κB, Other mechanisms whereby (A-IV)HDLs may reduce NO bioavailability involve inhibition of the acute phase response and inhibition of oxidised LDL formation, which also reduces NO bioavailability (52–54).

It is additionally conceivable that lipid-free and lipid-associated apoA-IV have endogenous anti-oxidant properties that reduce endothelial expression of ICAM-1 and VCAM-1 (18, 19, 55, 56). This possibility is based on the observation that TNF-α generates reactive oxygen species (ROS) and upregulates ICAM-1 and VCAM-1 expression in endothelial cells (57–59) and that HDLs protect against glucose induced ROS generation in endothelial cells (60). However, whether HDLs, and apoA-IV in particular, inhibit TNF-α induced ROS endothelial production is unknown and clearly worthy of further investigation.

In conclusion, this study demonstrates for the first time that apoA-IV potently inhibits vascular inflammation *in vivo* and *in vitro* and that the mechanistic basis of this effect is driven by inhibition of NF-κB activity and upregulation of the anti-oxidant and anti-apoptotic enzyme DHCR24. One of the most important observations to emerge from this study is that very low concentrations of apoA-IV are profoundly anti-inflammatory *in vivo*. While this finding identifies apoA-IV as being of potential therapeutic interest, the full-length apolipoprotein is unlikely to be a viable treatment option for inflammatory disorders. Future studies mapping the domains of apoA-IV that are responsible for its anti-inflammatory effects could, however, be further progressed, leading to the production of peptides that mimic this function and are potentially of considerable therapeutic value.

## Author Contributions

KS, K-AR, and JT designed the study and interpreted the results. KS and JT performed the experiments. KS, K-AR, and BC prepared and edited the manuscript. All authors contributed to the article and approved the submitted version.

## Conflict of Interest

The authors declare that the research was conducted in the absence of any commercial or financial relationships that could be construed as a potential conflict of interest.

## Publisher’s Note

All claims expressed in this article are solely those of the authors and do not necessarily represent those of their affiliated organizations, or those of the publisher, the editors and the reviewers. Any product that may be evaluated in this article, or claim that may be made by its manufacturer, is not guaranteed or endorsed by the publisher.

## Footnotes

1
https://imagej.nih.gov/ij/

2
www.graphpad.com

## Abbreviations

Apo: apolipoprotein
VCAM-1: vascular cell adhesion molecule 1
ICAM-1: intercellular adhesion molecule 1
DHCR24: 3 β -hydroxysteroid- Δ 24 reductase
HCAECs: human coronary artery endothelial cells
TNF- α: tumour necrosis factor alpha
I κ B α: I kappaB alpha
NF- κ B: nuclear factor-kappaB
NZW: New Zealand White.
