# Supplementary material for: Inhibition of Vascular Inflammation by Apolipoprotein A-IV
Source: Front Cardiovasc Med. 2022 Jun 30;9:901408. doi: 10.3389/fcvm.2022.901408 (PMC9279673; doi:10.3389/fcvm.2022.901408)
Supplement: Supplementary file 1 [file Data_Sheet_1.pdf]

Supplemental Information

Inhibition of Vascular Inflammation by Apolipoprotein A-IV

Kate Shearston[1], Joanne T.M. Tan[2], Blake J. Cochran[1], Kerry-Anne Rye[1].
[1]Lipid Research Group, School of Medical Sciences, Faculty of Medicine, University of New South Wales, Sydney, Australia; [2]Vascular Research Centre, South Australian Health and Medical Research Centre, Adelaide, South Australia.

Corresponding Author: Kerry-Anne Rye, Level 4E, Wallace Wurth Building, School of Medical Sciences ,Faculty of Medicine, University of New South Wales Sydney, Australia 2052.

Supplementary Table 1: Real-time PCR Primers

Gene	Direction	Primer sequence
**Human ICAM-1**	Sense	5’ CCA TCT ACA GCT TTC CGG CGC 3’
	Antisense	5’ CTC TGG GGT GGC CTT CAG CA 3’
**Human VCAM-1**	Sense	5’ ATG TAG TGT CAT GGG CTG TG 3’
	Antisense	5’ GGA ATG AGT AGA GCT CCA CC 3’
**Human MCP-1**	Sense	5’ TCC ACA ACC CAA GAA CAC A 3’
	Antisense	5’ TCC ACA ACC CAA GAA CAC A 3’
**Human DHCR24**	Sense	5’ CTC CTG CCG CTC TCG CTT ATC 3’
	Antisense	5’ GTC TTG CTA CCC TGC TCC TTC C 3’
**Human HRPT-1**	Sense	5’ TGA CAC TGG CAA AAC AAT GCA 3’
	Antisense	5’ GGT CCT TTT CAC CAG CAA GCT 3’
**Human B2M**	Sense	5’ CAT CCA GCG TAC TCC AAA GA 3’
	Antisense	5’ GAC AAG TCT GAA TGC TCC AC 3’

Supplementary Table 2: A single apoA-IV infusion does not affect plasma lipid, NEFA, apoA-I or apoA-IV levels in NZW rabbits

Plasma Lipids	Treatment	Baseline (T=0 h)	Collar insertion (24 h)	Euthanasia (48 h)
Apolipoprotein (µmol/L)	Saline	417.5 ± 35	412.2 ± 28	488.4 ± 35
	ApoA-I	415.6 ± 52	444.6 ± 61	448.5 ± 80
	ApoA-IV	359.6 ± 15	372.2 ± 26	456.9 ± 31
Phospholipid (µmol/L)	Saline	842.3 ± 37	856.7 ± 126	789.0 ± 36
	ApoA-I	904.0 ± 80	853.2 ± 87	887.8 ± 76
	ApoA-IV	843.0 ± 70	819.6 ± 87	887.4 ± 73
Unesterified Cholesterol (µmol/L)	Saline	144.7 ± 17	123.4 ± 10	135.5 ± 11
	ApoA-I	145.2 ± 8	155.2 ± 25	144.2 ± 6
	ApoA-IV	146.7 ± 11	147.4 ± 24	162.7 ± 14
Total Cholesterol (µmol/L)	Saline	769.1 ± 66	635.8 ± 50	704.5 ± 50
	ApoA-I	699.1 ± 74	727.3 ± 99	635.3 ± 83
	ApoA-IV	685.5 ± 75	685.5 ± 75	755.1 ± 68

NZW rabbits (n=6/group) were randomised to receive iv saline, lipid-free apoA-I (8 mg/kg), or lipid-free apoA-IV (1 mg/kg) at 24 h prior to placement of a non-occlusive, peri-arterial collar around the left common carotid artery. The animals were euthanised 24 h after collar placement and plasma lipid, apoA-I and apoA-IV levels were quantified as described in Materials and Methods.

Supplementary Table 3: A single apoA-IV infusion does not affect HDL composition in NZW rabbits

Treatment	PL/UC/CE/apoA-I) (*mol/mol*)	Particle Diameter (*nm*)
**Saline**	17.6±4.0/1.5±0.8/13.6±0.3/1.0	8.9
**ApoA-I**	21.8±3.0/1.6±0.5/11.0±2.0/1.0	9.0
**ApoA-IV**	19.5±6.0/1.8±0.2/10.6.0/1.0	9.2

NZW rabbits (n=6/group) were randomised to receive iv saline, lipid-free apoA-I (8 mg/kg iv), or lipid-free apoA-IV (1 mg/kg iv) at 24 h prior to placement of a non-occlusive, peri-arterial collar around the left common carotid artery. The animals were euthanised 24 h after collar placement and HDLs were isolated by ultracentrifugation and their composition was quantified as described in Materials and Methods.
